# Post-Operative Tetanus with Report of a Case1Read at the forty-second annual meeting of the Medical Association of Central New York, held at Auburn, October 19, 1909.

**Published:** 1910-01

**Authors:** M. M. Lucid

**Affiliations:** Cortland, N. Y.; Surgeon to Cortland Hospital


### Post-operative Tetanus with Report of a Case[1]

By M. M. LUCID, M. D., Cortland, N. Y.

Surgeon to Cortland Hospital.

THE subject of post-operative tetanus, with the report of a case, was suggested by hearing a scholarly paper on the subject read by Dr. Rudolph Matas, of New Orleans, before the American Surgical Association at the meeting held in Philadelphia, June, 1909, in which he considered from his personal experience with cases of post-operative tetanus (two in his own practice), the evident relation existing between the eating of uncooked vegetables, and the occurrence of post-operative tetanus appearing after operations in regions in close relation with the alimentary tract, or surfaces liable to fecal contact. It is, therefore, the purpose of this paper to discuss some of the phases of post-operative tetanus, with the report of a case occurring in my own practice, which, to all clinical appearances, adds another drop to the Matas bucket of prophylaxis, with the desire to urge upon the profession in general the very life-saving value of the prophylactic dose of antitetanic serum.

According to the literature at my command upon this subject, many patients apparently have been rescued from early graves by the timely administration of remedies of doubtful value, the recoveries more often getting into the literature than the deaths and, while the great aggregate in numbers are fatal, still the sum total of both the recoveries and deaths is not so large as to make the report of a single case superfluous.

I desire to mention at this time that in the discussion on tetanus at the District Branch Meeting, held in Syracuse in 1907, I made brief references to this same case, which I have deferred for publication till the present time.

CASE I. The case in question was referred to me for operation by the family physician, Dr. McBirney, of Willett, N. Y., November 21, 1905. History: Miss M., age 21, a vegetable cook in a hotel, with no history of any hereditary or chronic disease in the family. Personal history: until twelve months previous she enjoyed excellent health. Her present illness began ten months ago with symptoms first referable to the pelvis, and

---

1. Read at the forty-second annual meeting of the Medical Association of Central New York, held at Auburn, October 19, 1909.

later to the abdomen, resulting in a few months in a typical case of tubercular peritonitis with the primary focus undoubtedly of tubal origin.

She was admitted to Cortland Hospital, November 25, 1905, and kept under close observation till December 2, 1905, the date of the operation. A median incision was made, and an anomaly not commonly found, was discovered in the adherence of the bladder to the parietal peritoneum to within two inches and a half of the umbilicus. The amount of fluid free within the abdominal cavity was approximately one pint; the pelvic as well as the abdominal peritoneum was studded with bead-like tubercular deposits, showing secondary changes; while the right ovary and tube were extensively diseased, manifestly the primary focus of the tubercular deposit. The vermiform appendix presented distinct evidences of disease and was removed by the usual linen, purse-string method.

A right salpingo-oöphorectomy was performed, using iodine catgut, prepared by the Bartlett method. Numerous mesenteric glands were found enlarged. The closure of the abdominal incision was by the layer method, using iodine catgut, silkworm-gut, and linen for skin approximation. The operation was of brief duration, and, as is our custom, the strict observance of surgical asepsis. Two other abdominal operations were performed the same day under similar circumstances, both making uneventful recoveries.

The case presented no unusual features until December 8, six days after the operation, when in the evening she complained of pain in her back and stiffness of her jaws, both of which were but temporary. The silkworm was taken out and the wound appeared to be in healthy condition. The highest pulse rate recorded previous to operation was 117, the highest temperature, 101.2, which became 90 and 98, respectively, on the second day after admission.

The highest pulse rate recorded after operation, to December 8, was 95, the highest temperature, 100°, the day following the operation, both of which dropped to 84 and 99 respectively, on the second day following the operation. Later in the evening of December 8 the patient had some clonic twitching of the face, the nurse attributing this to hysteria.

I was called to the hospital at 1 A. M., December 9, finding the patient was unable to swallow the nourishment she had taken freely up to this time. The temperature was 101.2, the pulse 95. From the symptoms presenting, and the history of the case, the diagnosis of tubercular meningitis was entertained. Tetanus was thought of, but with no degree of certainty. During the night the patient slept but little, although she was quiet.

At 8 A. M., December 9, the stiffness of the jaws, with marked rigidity of the muscles of the back and neck, were distinctly in evidence. and there was no further time lost in searching for a positive diagnosis—post-operative tetanus. The wound was freely opened, but only a trace of bloody serum oozed out, bacteriological examination of which showed no tetanus bacilli; the least external stimulus, however, such as closing the door or touching the bed caused violent spasms.

Antitetanic serum was ordered but unfortunately failed to reach us. The patient grew steadily worse, crying out with abdominal cramps, clonic spasms of the face, arms and limbs, with more and more stiffness of the muscles of the back, until opisthotonos was pronounced, only the head and heels touching the bed. The pulse became weaker, the mind remained clear, the respiration more slow, till the patient died of cardiac failure at 6 A. M., on the eighth day following the operation, or thirty-four hours after the onset of the tetanic symptoms. The highest pulse and temperature recorded since the onset of the tetanic symptoms was pulse, 135; temperature, 103°. No post mortem was allowed.

Treatment of the case: The details of the treatment of this case, aside from giving magnesium sulphate and local wound treatment, are, in the light of our present knowledge sadly disappointing; and, while the diagnosis in this, as in most cases, was extremely easy and certain, still death ensued before we were able to procure antitetanic serum. We are not thus handicapped today, as the Health Department compels the local health officer to keep serum from the State Laboratories continually on hand, and as time goes on we see more and more indications for its use.

After a painstaking and careful analysis of all the facts in this case, we are confronted by a condition, which has not been satisfactorily accounted for, notwithstanding our advanced knowledge of the pathology of tetanus, (which is the key to the situation so far as treatment is concerned), since the discovery of the tetanus bacillus in 1884 by Nicolaier, and the isolation by Kitasato in pure culture in 1889. And that condition is the origin and regional distribution of accidental and post-operative tetanus in concealed parts of the body, protected from surface exposure, in which the rules of strict surgical asepsis have been observed.

From the fact that the tetanus bacilli were not shown from the culture made of the bloody serum taken from the wound on December 9, seven days after the operation, does not by any means preclude the existence of the tetanic infection in the wound, as the bacilli may remain at the point of infection frequently in such small numbers as not to be demonstrable, at the same time an enormous production of toxines are being injected into the circulation and at the time it may be impossible to find them in the circulation or remote organs.

On the other hand, the occasional post-operative death from tetanus, which occurs in the practice of experienced and competent surgeons, quite clearly point to another source of danger, which is not dependent upon faulty technic or imperfectly sterilised suture material, but to other sources of infection outside of, and apart from, the operation per se.

According to Matas and others, it is the direct contamination of the alimentary canal and its contents with living drumstick bacilli and their spores, swallowed in raw, uncooked vegetables, which correspond with the laboratory findings in regard to the vegetables found most commonly contaminated with tetanus germs and spores, such as celery, lettuce, cabbage, and the like, which are cultivated on manured soil and eaten raw or in a contaminated state. It is also a fact, according to Pizzini, that 5 per cent. of all individuals harbor the tetanus bacillus, or its spores, in an active state in the intestinal canal, and it may reach 20 per cent. in stable men or those working in dairies.

In view of this fact, the writer realises that the normal defenses of the body against intestinal infections are, in healthy individuals, amply able to protect them, even though the living tetanus bacilli be introduced into the alimentary canal with food, and the only manner in which we can account for the great numbers who survive, when operations are performed in the fecal contaminated areas, is through the anerobic character of the tetanus bacilli, plus the influence of the protective mechanism of the body, which very largely neutralises the virulent infections of the alimentary canal. In view of the above facts and in accordance with the preceding statements, the writer is of the opinion that it is not without the realm of possibility, in conditions of lowered vitality, in which the defensive power of the peritoneum is reduced to a minimum, as in tuberculous peritonitis, to have the tetanic infection emanate directly from the intestinal canal when it is opened, and be engrafted, during operative manipulation (more especially where sewing is done on the intestine), or after the amputation of the appendix, to the mechanically exposed or denuded surfaces within the abdominal cavity.

To obviate this possibility, prophylactic measures would be more than justifiable, even though tetanus occurred only once in 10,000 or 15,000 operative cases, to save the patient from almost certain death, when this form of inoculation occurs after operation. This antitetanic preparation consists of purging with castor oil three or four days before operation, the withholding of all raw and uncooked food, especially garden vegetables and fruit. In emergency cases, where dietetic preparation is not possible, an immunising dose of tetanus antitoxin is injected at the time of the operation. It has been our custom for some time to use the immunising dose of 1,500 units of tetanus antitoxin in gunshot wounds, compound fractures, and any injuries offering the slightest possibilities of tetanic infection, thus giving the patient the benefit of the doubt.

### PATHOLOGY OF TETANUS.

An understanding of the pathology of tetanus is the key to treatment. The origin is strictly anerobic, and one of the few anerobes that are known to be pathogenic to man. Wound conditions of such a character that infectious material is carried into the tissues, to the exclusion of oxygen, are necessary for the development of the germs. Tetanus organisms are not propagated by passing from case to case, but they spend the greater part of their career outside of the human body in some other host, or as harmless saprophytes. They are notoriously resistant; heating to 80° centigrade does not kill the spores; the bacilli survive boiling for one minute, the spores live indefinitely in the dry state. Therefore considering its ubiquity, its resistance, and its virulence, there is only one factor that prevents the disease from becoming epidemic—namely, its anerobic character.

Diphtheria toxin circulates in the blood, and is accessible to serum injections, as shown by the ideal results of antitoxin treatment. Tetanotoxin, on the other hand, does not continue to circulate free to any extent, but early becomes inaccessibly combined with certain nervous elements and cannot be reached by any known remedy, no matter how administered. That is the reason why the antitoxin, though proven an antidote in the laboratory, has been so disappointing clinically. No matter how administered, it cannot reach the toxin to neutralise it. Though the number of bacilli in the wound may be small, the amount of toxin produced is relatively great. The latter is remarkably poisonous; one three-hundreth of a grain is a fatal dose for a man (Brieger).

The toxin diffuses through the tissues around the point of infection, and Meyer and Ransom have shown how it enters the end of the motor neuron where it is not provided with a myelin sheath. The myelin sheath offers an absolutely impervious insulation, impenetrable alike both by the toxin and antitoxin. The toxin cannot enter an injured nerve save at its termination. neither can the antitoxin. Gumprecht proved that the motor nerves, and not the sensory, are the conductors of the toxin to the cord and medulla. Nor does it pass up in the lymph spaces about the nerve, but always by way of the axis cylinders. On reaching the cord, it enters into a union with the motor ganglion cells. By way of the commissure it diffuses to the other side of the cord, thence it passes upward along the motor tracts.

As the toxin is known to reach the cord by way of the motor nerves exclusively, is it not natural to infer that such portion of it as passes up a short nerve will reach the ganglia first, and produce the first symptoms? Thus, before the poison can reach the cord by way of the long nerves in the extremities, another portion of the same has entered the circulation, found access to the endings of a short motor nerve, like the inferior maxillary branch of the trifacial, reached the cord first and elicited the first symptom—namely, trismus. This looks like a plausible theory to explain the occurrence of trismus as the first motor symptom, no matter where the primary infection is situated on the body. This is in harmony with the clinical findings in local forms of tetanus, involving a single extremity, or in head tetanus. In local tetanus, the infection is of a mild character; there is enough toxin produced to involve a section of the cord through the motor nerves leading from the wound, but not enough to get into the general circulation, at least not for a relatively long period. Thus, we have in this form of tetanus primarily an involvement of certain muscle groups in the extremities, with trismus and general spasms later, if at all. The period of incubation is long, because the toxin all has to pass up some long nerve in an extremity, and not enough gets into general circulation to make the short cut mentioned above. These local cases are mild as a rule, and generally all recover.

The period of incubation in tetanus represents the time required for the bacilli to produce their toxin, and for the toxin to reach the cord and evoke the first motor phenomena. The longer the period of incubation, the milder will be the type of the disease.

The toxin enters into some sort of a firm combination with the nerve cells of the anterior horn and nuclei of the medulla. Whether this is due to its affinity for the cholesterin constituent of the cell is an open question. At any rate once the union has taken place, there is no remedy able to break it up and neutralise the toxin even if it could be reached by the antitoxin, and it has already been shown that this is not possible. The axis cylinders and motor nerve cells are as effectively out of reach of the antitoxin circulating in the tissue juices, as are the wires of the Atlantic cable insulated from the salt water. To facilitate classification, tetanus is divided into acute and chronic forms. Acute cases have an incubation of ten days or under, and chronic, or subacute, cases have a latent period of ten days or over.

TREATMENT OF TETANUS.

The available tetanus statistics, from the very nature of the subject, are unreliable, and show a lack of uniformity. Tetanus is comparatively rare after all, and no one observer has had enough cases in a year or series of years available for purposes of comparison under like conditions. Series of cases are collected from scattered reports, often incomplete. Recoveries will get into the literature oftener than deaths. Chronic cases that get well anyway are hailed as recoveries due to some special method of treatment.

The characteristic feature of tetanus is, that by the time we have made the earliest possible clinical diagnosis from the appearance of rigidity, it is already practically too late for treatment. Every motor nerve in the body is loaded with toxin, and conveying it to the cord. That means that if there is a fatal dose then on the way in these various nerves, no treatment, no matter what it is, can save life. Aside from prophylaxis, then, what is the problem?

PROPHYLACTIC TREATMENT.

As a prophylactic remedy tetanus antitoxin is conceded to be of undisputed value, as proven by animal experimentation, and in practice. While tetanus cannot thus be invariably averted yet, if it does develop, the period of incubation will be longer, and the cases are generally mild, and recover. It is needless to add that to be most effective, the immunising doses must be injected at once after injury.

41 MAIN STREET.

